# Behavioral assessment of pain in rodents: advances from evoked responses to spontaneous states and multimodal approaches

**DOI:** 10.3389/fpain.2026.1739384

**Published:** 2026-02-09

**Authors:** Meiqi Li, Yongxin Bao, Mingsen Chen

**Affiliations:** 1 Department of Physiatry, General Hospital of Northern Theater Command, Shenyang, China; 2 School of Acupuncture and Tuina, Liaoning University of Traditional Chinese Medicine, Shenyang, China

**Keywords:** behavioral assessment, pain, spontaneous pain, evoked pain, multimodal evaluation

## Abstract

Pain is widely recognized as a leading global health problem that markedly diminishes quality of life. Although assessment lies at the core of pain medicine, robust quantification remains difficult. In preclinical research, commonly used behavioral assays often blur the distinction between spontaneous pain and stimulus-evoked responses. Here, we review recent advances, clarify the conceptual and operational boundaries between spontaneous and evoked pain, and provide a multidimensional comparison of major traditional behavioral paradigms. To address shortcomings in objectivity and reproducibility, we also summarize emerging evaluation strategies. Finally, leveraging bioinformatics and machine learning, we identify pain-associated metrics and propose building multimodal datasets and AI-driven feature-extraction pipelines to enhance the translational value of animal data for clinical pain research.

## Graphical Abstract

**Figure:**
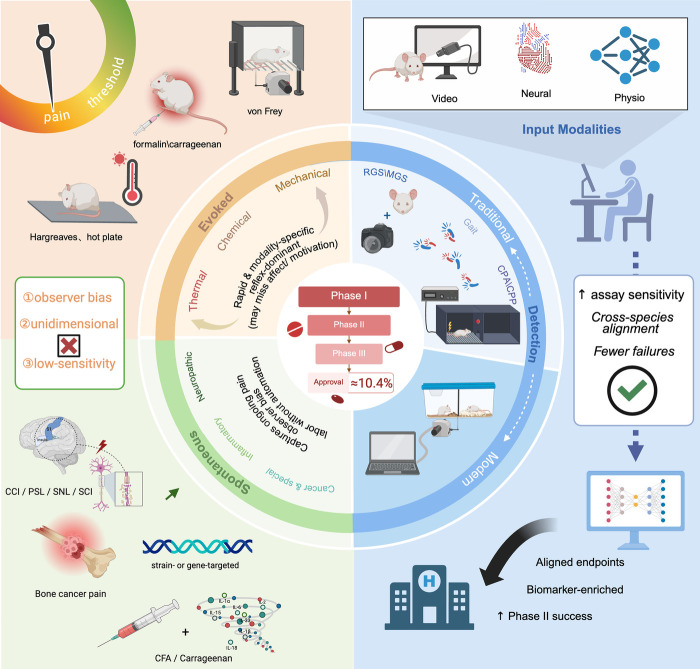
Created in BioRender. i, L. (2026) https://BioRender.com/ab5yjcf.

## 1 Introduction

Pain is a distressing sensory and emotional experience elicited by noxious stimuli and expressed through affect and behavior. The International Association for the Study of Pain (IASP) defines it as “an unpleasant sensory and emotional experience associated with, or resembling that associated with, actual or potential tissue damage” (1). In recent years, pain management has been shifting from symptom control to precision care. Developing more accurate and objective assessment tools—and applying comprehensive, multidimensional evaluations—is essential for elucidating pain mechanisms and for rational design of targeted analgesic interventions.

As the critical bridge between preclinical research and clinical application, behavioral assessment in experimental animals faces two major challenges. First, because pain arises from distinct mechanisms, its stimulus-evoked responses and spontaneous states also differ. Distinguishing these states is pivotal for understanding neuroplastic changes, phenocopying clinical pain, and evaluating analgesic efficacy. Multiple reviews and experimental studies suggest that a preclinical focus on evoked pain to the neglect of spontaneous pain undermines clinical relevance and translatability (2, 3). Over the past decade, the overall success rate of pain therapeutics progressing from Phase I to approval has been ∼10.4%; cross-therapeutic analyses indicate that conflating these two states likely contributes to evaluation bias and translational failure (4). Second, many traditional assays rely on reflexive endpoints or single-dimension observations and are susceptible to observer effects, limiting objectivity and reproducibility.

Beyond pain-focused studies, all animal experiments carry an ethical imperative to prevent unnecessary pain. In accordance with the 3Rs (Replacement, Reduction, Refinement) and the ARRIVE reporting guidelines, investigators should prioritize low-invasive, low-stress assessments. Doing so is not only a scientific, legal, and moral obligation; it also reduces variability and poor reproducibility arising from stress-related alterations, diminishes “pseudo-analgesia/pseudo-exacerbation” bias, and improves cross-species comparability.

Rodents, with high behavioral responsiveness and translational relevance, remain the most widely used species in preclinical pain research (2). Although several reviews have summarized rodent pain assays, the present work focuses specifically on the core challenge of differentiating spontaneous from evoked pain to provide a more integrated view. We begin with mechanisms underlying these states and map them onto commonly used rodent behavioral paradigms, analyzing strengths and limitations of each. We then look forward, tracing the evolution from classical readouts to AI-enabled multimodal analytics and considering their implications for translation. This framework not only opens avenues for methodological innovation and greater precision in preclinical assessment, but also advances animal welfare and guides the development of related human clinical applications.

## 2 Stimulus-evoked pain

**Figure 1:**
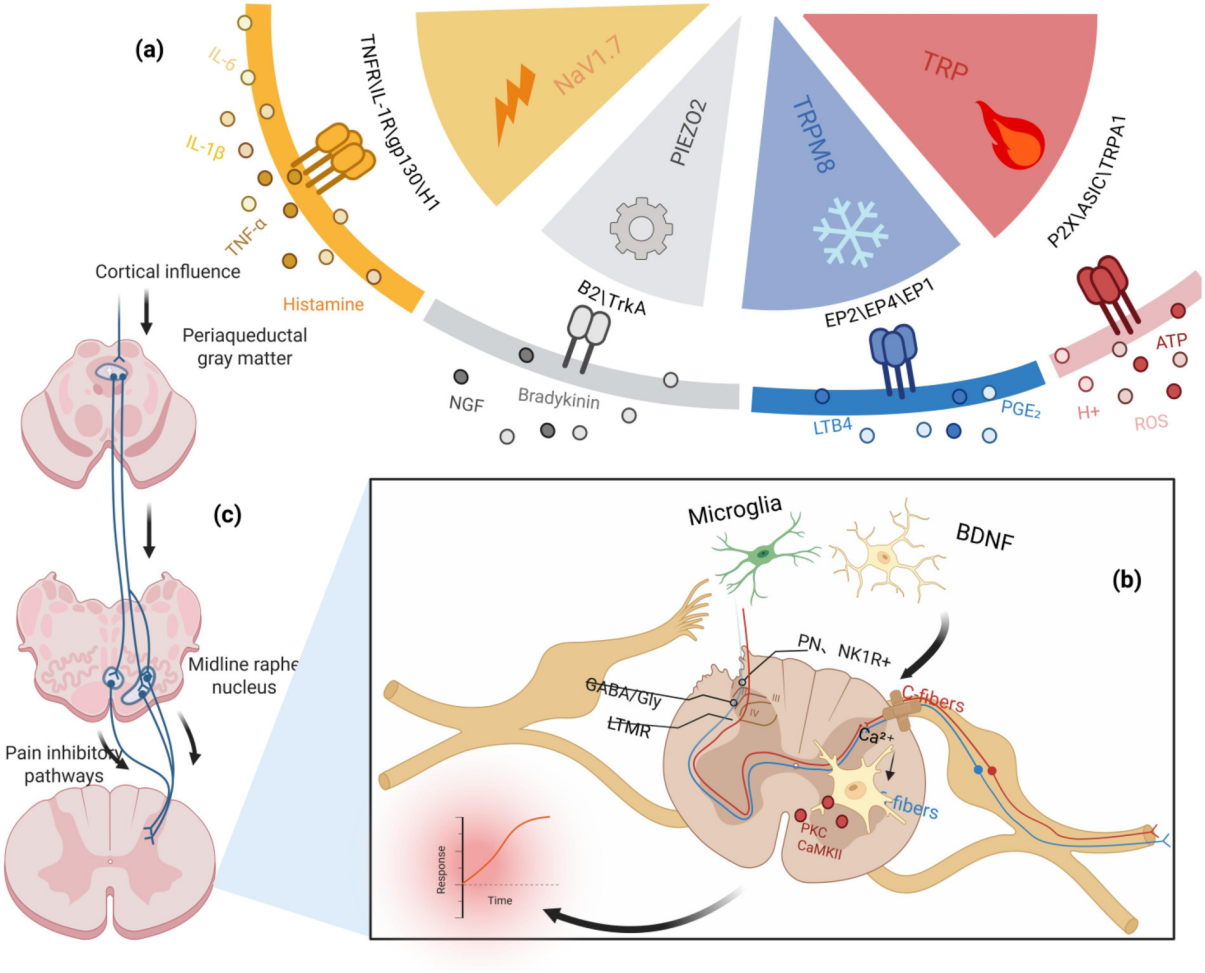
Mechanisms of evoked pain. **(a)** Peripheral sensitization: Injury/inflammation releases TNF-α, IL-1β, IL-6, histamine, bradykinin, NGF and eicosanoids (PGE₂/LTB₄), which act via TNFR/IL-1R/gp130/H1, B₂/TrkA and EP2/EP4/EP1 to lower nociceptor thresholds by PKA/PKC-dependent modulation of NaV1.7, TRP (incl. TRPA1), TRPM8, PIEZO2, ASIC and P2X channels; extracellular ATP, H^+^ and ROS further drive depolarization. **(b)** Dorsal horn circuit remodeling: Potentiated C-fiber input induces Ca^2+^ influx and PKC/CaMKII activation, while microglia-derived BDNF downregulates KCC2 causing GABA/glycine disinhibition; misrouting of low-threshold Aβ afferents engages lamina I–II and excites NK1R^+^ projection neurons, producing central sensitization and allodynia. **(c)** Altered descending control: Cortical influences dysregulate the PAG–midline raphe pathway, reducing serotonergic/noradrenergic inhibition and/or enhancing facilitation, thereby sustaining spinal hyperexcitability and evoked pain.

Stimulus-evoked pain primarily denotes hyperalgesia to noxious stimulation and allodynia to innocuous tactile input. Its emergence is typically driven by peripheral sensitization, dorsal horn circuit remodeling, and altered descending modulation (5–7) (Figure 1). In preclinical research, evoked assays remain the most commonly used approach because stimulus delivery is readily controlled and behavioral outcomes are straightforward to quantify. However, these measures capture only part of the pain construct and can be influenced by supraspinal integration and affective state. Therefore, complementary central readouts and non evoked measures are needed to provide a more complete assessment, as discussed below.

### 2.1 Mechanical stimulation

Mechanical stimulus–evoked pain is the most widely used assay class in animal studies. These methods apply calibrated force to sensitive sites using dedicated tools—fine pins, von Frey filaments, or pressure probes—to elicit nocifensive behaviors (e.g., paw withdrawal, licking/guarding, escape), thereby quantifying mechanical sensitivity and determining pain thresholds (8, 9). Beyond the limbs and tail, tactile allodynia within trigeminal territories is often assessed by probing the periorbital region (10) or the whisker pad, whereas in visceral pain paradigms, referred somatic mechanosensitivity can be quantified by applying von Frey filaments to the abdominal wall or other truncal sites (11).

Originating from clinical monofilament testing and standardized for rodents, the von Frey filament method is the workhorse for determining mechanical thresholds. A series of calibrated filaments is applied in ascending or descending order, and the 50% paw withdrawal threshold (PWT) is estimated using the Dixon up–down method or the SUDO fixed-trial algorithm (12–14). Algorithmic refinements by Christensen and colleagues further improved efficiency and precision by computing thresholds from the exact filaments used and their spacing (15). Subsequent electronic von Frey (eVF) devices replace manual force delivery with an electronic transducer, enabling more precise stimulation and automated recording of force slope and withdrawal thresholds via software (9). Compared with manual testing, the standardized stimulus delivery and automated readout reduce experimenter bias and support higher-throughput testing across multiple time points and larger cohorts (16, 17).

### 2.2 Electrical stimulation

Electrical stimulation provides a precisely controllable noxious input and is commonly used in rodents to quantify evoked pain and to evaluate the functional status of sensory afferent fibers. In classic grid- or plantar foot-shock test, the current intensity is increased in a stepwise manner while pain-related behaviors such as jumping and vocalization are observed to derive behavioral thresholds for nociceptive sensitivity, these tests have also been used to demonstrate pharmacological sensitivity to analgesics (18).

Building on this concept, sine-wave transcutaneous electrical stimulation has been applied to the hind paw, and stimulation at 5, 250, and 2,000 Hz is used to preferentially weight responses associated with C-, Aδ-, and Aβ-fiber activity, respectively. Pain intensity can then be indexed by endpoints such as paw withdrawal or vocalization (19). Studies have shown that varying stimulation frequencies can preferentially recruit distinct classes of afferent fibers. Accordingly, sine-wave transcutaneous electrical stimulation is able to detect shifts in nociceptive thresholds under inflammatory and postoperative pain conditions, and it can capture the analgesic effects of opioid treatment (20). Methodological advances, including Neurometer-based threshold measurements (21) and automated vocalization-threshold assays (22), have improved the efficiency and standardization of sine-wave–based assessments.

In anesthetized animals, electrical stimulation can also evoke flexor/withdrawal reflexes; motion sensing and electromyography (EMG) allow separation and quantification of Aδ- and C-fiber–related components (23). Moreover, in a clinical study, Tomoya compared the effects of magnetic stimulation, bipolar electrical stimulation, and monopolar electrical stimulation on the human flexor reflex and pain perception, supporting the translational relevance of electrical stimulation–based paradigms (24). Frequency-specific electrical thresholds have been increasingly used to phenotype sensory abnormalities across disease models and to interrogate peripheral mechanisms that regulate tactile and nociceptive sensitivity under physiological and pathological conditions (25, 26). In studies interrogating visceral pain related pathways, focal electrical stimulation can be applied to the rectum or colon to elicit visceral responses (e.g., visceromotor reflex activity) (27). Finally, repetitive nociceptor stimulation is widely used to model temporal summation (“wind-up”) as an electrophysiological correlate of central sensitization (24).

Overall, the major strengths of electrical stimulation lie in the precise control of intensity, frequency, and pulse parameters, facilitating reproducibility and cross-laboratory standardization. However, stable electrode contact is required, and electrical stimuli do not directly mimic tissue injury or inflammation, so electrical paradigms are typically used as complementary outcome measures alongside mechanical/thermal/chemical assays rather than as a primary method for pain-model induction.

### 2.3 Thermal stimulation

Thermal stimulus–evoked pain is widely used to probe physiological mechanisms and pharmacodynamic effects in pain research (9, 28). In rodents, the plantar hind paw and tail are the most standardized testing sites. Controlled heat stimuli applied to these sensitive regions activate cutaneous heat sensitive nociceptors as temperature rises, thereby initiating nociceptive signaling from the periphery to the central nervous system. Thresholds are inferred from the temperature at first nocifensive behavior or from response latency, providing an index of thermal sensitivity (29).

The hot-plate test, first introduced by Woolfe in 1944 for analgesic screening, places a rat on an enclosed temperature-controlled plate and quantifies analgesia by the latency to escape-like behaviors (e.g., paw licking or vocalization) (30). However, unstable temperature control can induce stress, non-nociceptive movements during testing may confound scoring, and behavioral adaptation/learning can further bias results. The Hargreaves test, which delivers radiant heat to a localized plantar site, mitigates the temperature-control limitations of the hot plate (28, 31). Relative to hot-plate testing, Hargreaves offers: (1) precise control of target temperature and heating rate, reducing inter-individual variability during operation (32); (2) measurement under unrestrained conditions, minimizing premature activation of other physiological responses and reducing stress (33, 34); and (3) automated operation, which improves data reliability (35). Many rodent readouts do not translate directly to large animals. Although adaptations of the Hargreaves paradigm have been reported in dogs and non-human primates (36, 37), they require substantial modifications. Consequently, the method's technical complexity and equipment cost limit broader use in large-animal or translational settings (38, 39). Greater standardization of stimulus generation (explicit temperature control, defined heating slope, automated logging) and integration with brain-level readouts (e.g., fMRI, single-unit activity) may enhance the generalizability and translational value of thermal nociception assays (40).

### 2.4 Chemical stimulation

Injecting algogenic substances or inflammatory mediators into rodents elicits sustained, robust, and readily scored evoked pain behaviors. In the writhing test, dilute acetic acid is administered intraperitoneally (i.p.) to quantify abdominal constrictions and whole-body writhes, providing a sensitive assay for analgesic efficacy (41). Compared with mechanical or thermal paradigms, these responses typically last longer and are straightforward to quantify (42). The formalin test is more complex: subcutaneous injection of formalin into the paw induces prolonged pain (43). Using Dubuisson's scoring system, the post-injection response is parsed into two distinct phases: Phase I reflects acute activation of primary nociceptors and is a standard readout of evoked pain, whereas Phase II captures inflammation-driven activity and central sensitization, serving as an index of spontaneous pain. Accordingly, the test can evaluate the duration of analgesic effects and differentiate centrally acting drugs (e.g., opioids, which often suppress both phases) from peripherally acting drugs (which predominantly reduce Phase II) (44, 45).

Notably, skin properties vary substantially across anatomical sites (46, 47), and estimated nociceptive thresholds can be influenced by operator-dependent factors such as stimulation angle and contact area (48). In rodents, the plantar hind paw and tail are the most commonly tested sites for evoked assays and are widely used in inflammatory and peripheral nerve injury models (9). By contrast, stimulation of the periorbital region and the whisker pad offers greater anatomical specificity for trigeminal territories and is therefore commonly used in models of cephalic pain, including migraine and trigeminal neuralgia (49). In visceral pain paradigms, referred somatic hypersensitivity can be assessed at truncal sites. Accordingly, the test site should be chosen to match the model's primary pathway and its corresponding innervation territory; otherwise, pain severity may be under- or overestimated (50).

Multiple studies indicate that thermal assays offer greater biological sensitivity than mechanical tests and, owing to more standardized stimulus delivery, are well suited to detecting subtle shifts in hypersensitivity (51). However, they are more vulnerable to confounding by anxiety and related affective states (52). By contrast, mechanical stimulation maps more directly onto human neuropathic/mechanical pain, is not subject to central processing delays inherent to cutaneous thermoreceptor pathways, and thus may provide a more reliable translational benchmark (53). Compared with thermal or mechanical stimulation, electrical stimulation offers more pronounced advantages in precise control of stimulus timing and intensity and in its compatibility with neurophysiological readouts; however, similar to thermal assays, it is also susceptible to confounding factors (54). Chemical paradigms, relative to the three above, additionally capture aspects of spontaneous pain and therefore furnish a broader evaluative window. Clinically, the most prevalent pain phenotypes are associated with mechanical, chemical/inflammatory, and cold pain, with limited contribution from heat pain. Consequently, selecting assays that match the intended clinical context is critical for translation. A side-by-side overview of strengths, limitations, and translational alignment is provided in Table 1.

**Table 1:** Comparison of evoked pain preclinical models in rodents.

Method		Advantages	Limitations	Applicable contexts	Refs
Mechanical stimulation	Von Frey Filament Test	· Standardized procedures · High sensitivity · Can be automated (electronic von Frey)	· Susceptible to stress and environmental interference · Large inter-individual variability	· Neuropathic pain · Mechanical pain threshold testing	(9, 51, 55–61)
Thermal stimulation	Hot Plate Test	· Simple operation · Suitable for initial analgesic screening	· High stress response · Learning effects · Difficult to distinguish analgesia from motor suppression	· Acute thermal pain assessment · Analgesic screening	
	Hargreaves Test/Paw Withdrawal	· Precise localized thermal stimulation · Automated latency recording · High sensitivity	· High equipment cost · Unsuitable for large animals · Environmental factors can affect results	· Thermal hyperalgesia research · Acute/chronic pain	
Chemical stimulation	Writhing Test	· High sensitivity · Simple procedure	· Low specificity · Easily induces stress	· Visceral pain models · Analgesic screening	
	Formalin Test	· Distinguishes acute and persistent pain phases · Clear positive behavioral responses	· Significant tissue damage · Complex interpretation	· Inflammatory pain models · Chronic pain mechanisms	
Electrical stimulation	Flinch、vocalization、escape	· Precise parameter control · Good reproducibility · Simple setup; can be automated · Distinguishes short-latency (A-fiber–mediated) and long-latency (C-fiber–mediated) components	·Better for shock-sensitivity screening than pain phenotyping ·Not tissue-/inflammation-mimicking ·High variability	· Neuropathic pain · Chronic pain mechanisms · Central sensitization	
	visceral responses	· Probes visceral afferent pathways · Translational potential	·Standardization/specificity issues ·Less physiological	Visceral pain models	

## 3 Spontaneous pain

Spontaneous pain refers to ongoing or fluctuating pain that occurs in the absence of an identifiable external trigger (62). It is typically driven by persistent internal pathological or injury-related processes, such as ongoing inflammatory activity, injury-associated signaling, or ectopic discharges following nerve damage (63); when these internal drivers subside, spontaneous pain may correspondingly diminish. External stimuli may exacerbate its expression but are not required for its occurrence (64, 65). In contrast, chronic pain may persist even when no discernible triggering factor, whether external or internal, can be identified (66).

**Figure 2:**
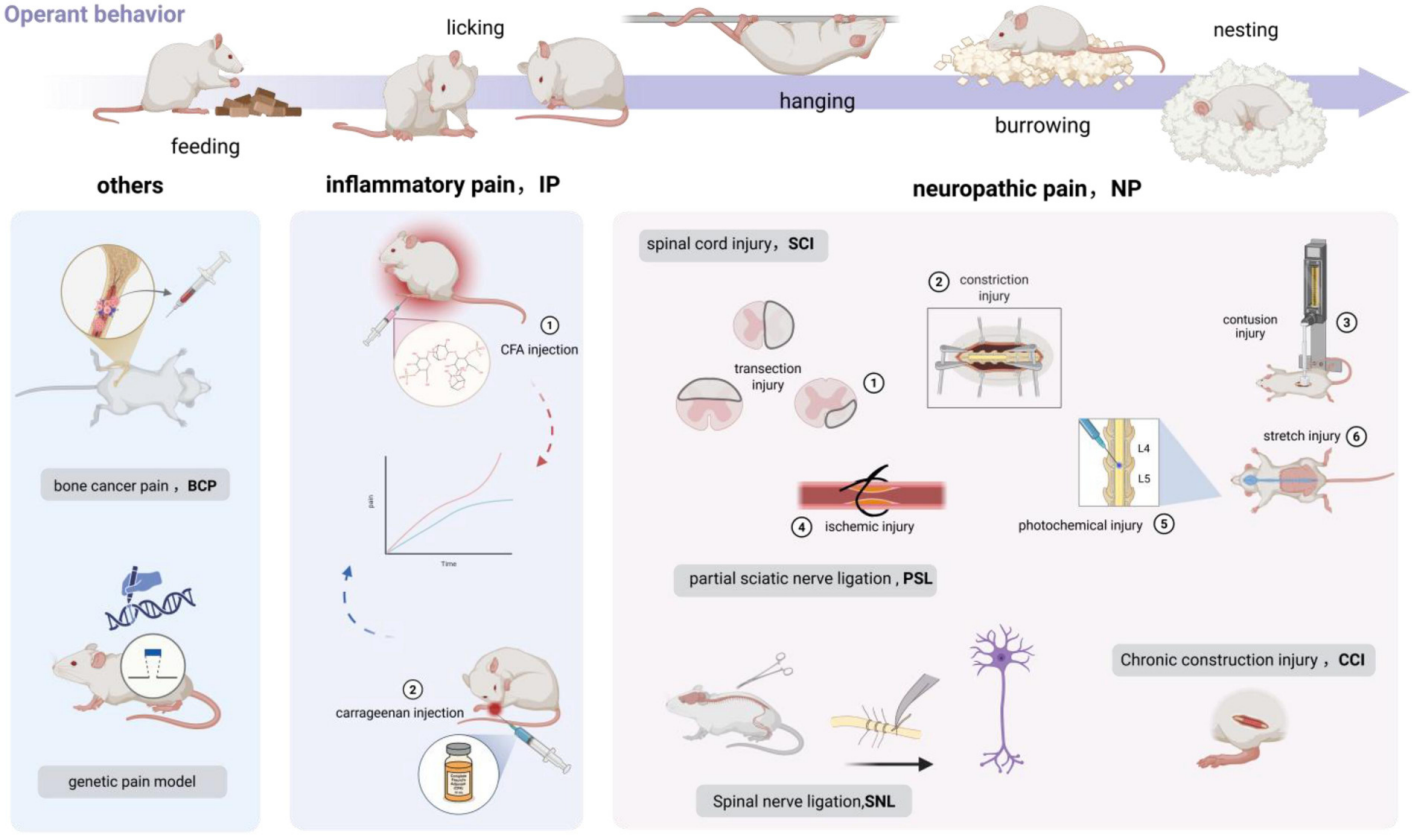
Spontaneous pain models linked to operant readouts.spontaneous pain arises after injury or dysfunction without requiring external stimulation. The top row defines the core operant/home-cage behaviors—feeding, licking, hanging, burrowing, nesting—that decrease during ongoing pain and rebound with analgesia. The three panels show disease paradigms that internalize spontaneous pain via ectopic activity and central sensitization: inflammatory (CFA, carrageenan), neuropathic (SCI—transection/compression/contusion/ischemic-photochemical/stretch; PSL/PSNL; SNL L5 ± L6; CCI), and other (bone cancer pain; genetic models). Across panels, these same behaviors serve as the primary non-evoked endpoints.

Unlike evoked pain, spontaneous pain is not generated by classical noxious stimulation *per se*. Accordingly, reliance on stimulus-evoked assays alone, without incorporating non-evoked outcomes, can misrepresent clinically relevant pathophysiology and reduce translational relevance (67, 68). At present, fully dissociating spontaneous pain from concomitant stimulus-evoked hypersensitivity remains challenging, and no standalone approach reliably produces “pure” spontaneous pain in the absence of evoked sensitization. In preclinical studies, spontaneous pain therefore most often emerges endogenously within disease-relevant models and is inferred from non-evoked behaviors. An overview of the relevant models and non-evoked readouts is provided in Figure 2.

### 3.1 Operant behaviors

Operant behaviors are regulated by limbic circuits—including the hippocampus, amygdala, and prefrontal cortex—which govern emotion, motivation, and goal-directed action (69). Persistent pain selectively disrupts these networks, suppressing motivation-driven spontaneous behaviors; accordingly, such behaviors are sensitive readouts of ongoing pain. In humans this suppression manifests as reduced locomotion and social engagement, whereas in rodents it appears as decreases in burrowing, nesting, feeding, and hanging (70, 71).

Liu et al. reported consistent locomotor suppression induced by neuropathic pain across rodent strains, observation windows, and pain types (72). Burrowing suppression not only signals persistent spontaneous pain but also differentiates neuropathic from inflammatory states (73). Hanging behavior under persistent pain is reversibly improved by analgesics and thus serves as a useful endpoint for preclinical pain studies and analgesic development (71). Beyond manual scoring, cross-species (>45 species) pose-estimation models have enabled video-plus-algorithm pipelines that identify defensive/protective actions under standardized stimulation, reducing subjectivity and improving reproducibility (74, 75); these advances underscore the cross-species translational promise of spontaneous-behavior endpoints (76). Quantifying spontaneous behavior is non-evoked and minimally intrusive, preserves innate protective responses, reduces procedure-induced stress, and aligns with the 3Rs ethical framework (77). Nonetheless, these readouts can exceed the granularity of single-score schemes; for example, Zhang et al. used video-based hanging analysis with the HomeCageScan platform to automate evaluation, demonstrating robust suppression across multiple chronic pain models with reversal by analgesics and notable translational potential (71). Finally, spontaneous-behavior measures may lag real-time physiology; integrating them with immediate physiological indices—such as telemetric heart rate/heart-rate variability and plasma corticosterone in rodents—can mitigate this limitation (78).

### 3.2 Neuropathic pain models

Many widely used preclinical models endogenously generate spontaneous pain. Neuropathic pain models constitute a major class; clinical evidence indicates that assessments based solely on evoked stimulation cannot reliably distinguish painful from painless neuropathy (79). Classic paradigms—spinal cord injury (SCI), chronic constriction injury (CCI), partial sciatic nerve ligation (PSL/PSNL), and spinal nerve ligation (SNL)—produce ongoing spontaneous pain through persistent ectopic discharge and central sensitization. Common limitations include between-batch variability from surgical trauma and operator technique, overreliance on evoked endpoints with poor sensitivity to spontaneous pain, and observer-dependent, low-throughput scoring.

#### 3.2.1 SCI

Thoracic (T8–T10) contusion, compression, or transection models induce post-injury ectopic activity, abnormal spinal and thalamo-cortical rhythms, and glial responses that together sustain neuropathic pain (80). Induction requires microsurgical exposure and standardized impactors (weight-drop or electronically controlled) to deliver reproducible parenchymal injury, followed by neurological and pain assessments (81). In 2017, the Louisville Injury System Apparatus (LISA) introduced displacement-controlled contusion in mice using laser range sensing plus software control, achieving highly consistent T10 injuries (82). Fully automated mouse contusion systems subsequently combined laser distance sensing with motorized stages and intelligent software to produce user-defined SCI severities with improved impact localization, parameter standardization, and operational ease (83). Finite-element (FE) models now map injury mechanics and parameter sensitivity, supporting device standardization and cross-platform comparability and enabling prediction of tissue stress/strain during trauma (84).

#### 3.2.2 PSL and SNL

These models use “loose” ligation to induce partial ischemia and/or demyelination of the target nerve, yielding both stimulus-evoked hypersensitivity and spontaneous pain. For SNL, tight unilateral ligation of L5 (±L6) spinal nerves in rats produces guarding/defensive behaviors on the injured side with concomitant neuroinflammation and glial activation (85). Pain severity is typically indexed by postoperative mechanical and thermal hypersensitivity together with injured-side spontaneous behaviors (86). By ∼4 weeks, the central amygdala shows enhanced astrocytic reactivity and altered neuronal excitability—implicating not only dorsal horn plasticity but also remodeling of ascending–limbic circuits (87); accordingly, facial grimacing and affect-related behaviors are increasingly used as endpoints for spontaneous pain.

### 3.3 Inflammatory pain models

Injection of Complete Freund's Adjuvant (CFA) or carrageenan is commonly used to establish inflammatory pain. CFA induces not only stimulus-evoked hypersensitivity but also persistent pain with disrupted spontaneous behaviors, and is therefore widely used to model chronic inflammation. Under anesthesia, CFA is typically delivered into the plantar paw, whisker pad, or joint cavity to elicit localized chronic inflammation; successful induction is characterized by sustained mechanical and thermal hypersensitivity alongside histopathologic inflammatory changes (88). Recent work has delineated dose-dependent effects of CFA on rodent behaviors and tissue pathology, providing guidance for protocol standardization. Notably, after CFA, animals exhibit reduced activity, alterations in spontaneous behavior, and increased facial grimace in the absence of external stimulation; these indices are reversed by analgesic interventions, supporting the presence of ongoing pain (89).

By contrast, carrageenan provokes short-lived but pronounced evoked hypersensitivity, with edema, thermal and mechanical hyperalgesia, and cyclooxygenase upregulation emerging within hours (88). Unlike CFA, carrageenan-associated spontaneous pain is transient and is therefore used primarily to model acute inflammation. Outcome measures for carrageenan include changes in spontaneous behaviors as well as affect-related assays such as the elevated plus maze (EPM) and the forced swim test (FST); however, concerns persist regarding observer subjectivity and the reliability of such evaluations (89). In addition, batch-to-batch variability in induction strength (dose, site, volume), the limited sensitivity of traditional threshold assays to non-evoked pain, and the subjectivity of manual scoring remain unresolved challenges.

### 3.4 Other pain models

#### 3.4.1 Bone cancer pain (BCP)

BCP features sustained pain as a defining characteristic. Intra-medullary injection of tumor cells into the femur or tibia produces stable osteolysis. Enrichment of inflammatory mediators and NGF drives peripheral sensitization with ectopic discharges, tumor-induced sensory sprouting, and neuroma-like remodeling (90). Within the dorsal horn, widespread central sensitization and heightened synaptic excitability convert persistent peripheral input into tonic pain output even at rest (91). Together, these peripheral and central alterations provide the anatomical and electrophysiological substrate for the spontaneous, unpredictable pain typical of BCP.

#### 3.4.2 Genetic models

Monogenic pain syndromes are modeled by knocking in/out human orthologs with defined variants: SCN9A/Nav1.7 gain-of-function (GOF) causing erythromelalgia, loss-of-function (LOF) causing congenital insensitivity to pain; TRPA1 GOF causing familial episodic pain syndrome; and mutations in NGF/TrkA producing hereditary sensory and autonomic neuropathies (HSAN). These models align closely with clinical phenotypes and thus offer strong translational value. Nonetheless, strain, sex, and age exert substantial influence. Pronounced sex differences have been reported in the molecular and cellular mechanisms of pain (92), implying that genotype effects may be sex-contingent. Both clinical and animal data indicate that aging reshapes the linkage between nociception and inflammation (93). Cross-strain resources and databases can help select the most appropriate strain/age/sex for a given design; multi-background genetic panels have been shown to quantify complex-trait variance systematically and improve reproducibility (94).

Across the above models, common limitations persist: batch-to-batch variability arising from surgical trauma and operator technique; heavy reliance on evoked endpoints with poor sensitivity to spontaneous pain; and observer-dependent, low-throughput scoring (Table 2). As early as 2011, investigators noted that conventional evoked assays overlook spontaneous pain, a core symptom in patients with spinal cord injury (SCI) (95). More recent work linking circuit dysfunction to pain has identified sleep and respiration as behavioral indicators after SCI (96). For example, post-injury spontaneous nociceptor discharges can fragment NREM sleep, whereas analgesic interventions restore sleep continuity (97). These findings indicate that spontaneous pain perturbs endogenous physiological rhythms, underscoring their cross-species promise as physiological readouts. Quantification of such spontaneous behaviors can be achieved noninvasively with home-cage monitoring systems; adding computer vision improves objectivity under unrestrained conditions (98), and integrating machine learning enables more efficient pain detection and grading (99).

**Table 2:** Algogenic mechanisms and appraisal of common spontaneous-pain–relevant models.

Model category	Classic model	Algogenic mechanism	Brief appraisal	Refs
Neuropathic pain	CCI	Loose ligatures around the sciatic nerve → mild compression/inflammation and ectopic firing	- Simple and robust; - Ligature tension is operator dependent - Increases between-batch variance	(100, 101)
	PSL/PSNL	Partial ligation of dorsal fascicles → focal ischemia/demyelination and persistent ectopic activity	- Models partial nerve injury - Duration/intensity vary with fiber proportion and suture material	(102)
	SNL	Tight ligation of L5 ± L6 spinal nerve → segmental axonal injury and central sensitization	- Fast-onset - Long-lasting - Technically demanding and affected by motor/affective confounds	(86, 103)
	SCI—contusion	Controlled impact causes parenchymal mechanical injury	- Clinically relevant - Parameter-standardizable - Motor/autonomic deficits confound behavior	(104)
	SCI—compression	Sustained external compression producing ischemia + mechanical injury	- Force and time window are controllable - Mechanism not identical to blunt contusion	(105)
	SCI—transection/hemisection	Partial or complete interruption of spinal tracts	- Pathway-specific - Highly invasive - Limited translational relevance	(106)
	SCI—photochemical/photothrombotic	Rose Bengal + illumination → focal thrombosis/ischemia	- Minimally invasive - Preciseprimarily models ischemia	(105)
	SCI—stretch	Rapid over-stretch injures axons/myelin	- Useful for acceleration–deceleration mechanisms; - Device/parameter standardization required	
Inflammatory pain	CFA	Local immune-inflammatory response → peripheral + central sensitization; suppression of spontaneous behaviors	- Stable chronic phenotype - Severity depends on dose/site/volume - Needs welfare oversight	(89, 107, 108)
	Carrageenan	Acute inflammation with COX/prostaglandin upregulation; transient ongoing pain	- Simple and fast - Limited for sustained spontaneous pain	(109, 110)
Other pain types	BCP	Intramedullary tumor → NGF enrichment and aberrant sprouting/neuroma; widespread dorsal horn sensitization	- High clinical relevance - Tumor heterogeneity	(111, 112)
	Genetic pain models	Channelopathy/neurotrophin variants (e.g., SCN9A, TRPA1, NTRK1)	- High clinical relevance - Phenotypes depend on strain/sex/age	(113, 114)

## 4 Assessment methods

At present, most preclinical studies rely on single dimension behavioral assays that chiefly interrogate evoked pain, typically quantified by reflex based withdrawal thresholds or latencies. Although these paradigms sensitively capture hypersensitivity and detect acute analgesic effects, they often miss ongoing spontaneous pain and the affective and motivational dimensions that dominate the clinical burden, especially in chronic pain. Clinically, spontaneous pain is tightly linked to supraspinal integration and impaired endogenous pain modulation (115). Descending brainstem control recruited through the periaqueductal gray and rostral ventromedial medulla can inhibit or facilitate spinal nociceptive transmission and provides a mechanistic basis for endogenous analgesia (116); in humans, conditioned pain modulation is widely used to probe this function and is often viewed as the behavioral counterpart of diffuse noxious inhibitory controls. Because many peripheral mechanisms are conserved but higher level modulation may differ across species (117), developing standardized spontaneous pain readouts with improved objectivity, reproducibility, and scalability is essential for translation. Table 3 provides an operational matrix to guide assay selection and study design.

**Table 3:** Operational matrix for spontaneous pain assays.

Method family	Assay/Platform	Primary endpoint(s)	Session &sampling	Cost & time burden	Confounders & controls	Automation readiness	Refs
Gait	ADWB/Static WB; CatWalk/DigiGait	Hindlimb load asymmetry; stride/stance/swing; paw intensity	2–3 days acclimation; speed-matched trials; ≥3 valid runs/animal	High equipment; Medium time burden (habituation + valid runs); Low manual scoring	Body mass & speed as covariates; runway lighting/contrast; strain/sex as strata or covariates	High	(119, 126, 201)
Facial expression	MGS/RGS; aMGS/aRGS (automated)	AU composite score (orbital, ear, nose/cheek, whisker); automated AU probability	5–30-min windows; multi-timepoint follow-up; fixed camera distance & lighting	Low equipment; High labor (manual scoring) unless automated; Short capture, long scoring	Rater bias → automation; head pose/occlusion; strain/sex differences	Med–High	(156, 202)
Affective–motivational	CPP/CPA	Δtime (preference/avoidance index; relief reward/pain aversion)	Standard 3-day protocol; clearly defined analgesia/pain pairing; counterbalancing	Medium equipment; High time burden (multi-day, manual handling); Medium analysis	Learning/stress/activity; sex effects; contextual cues (floor/visual/odor)	Med	(203, 204)
AI multimodal	PAWS (hi-speed kinematics)/Keypoint-MoSeq/Facemap/USV (DeepSqueak)/IRT	Composite SPI (weighted gait/face/USV/physiology); AUC/ICC	Continuous or batch capture; marker-less pose/face; cross-day calibration	High upfront cost (hardware/compute/annotation); Low per-animal labor once deployed; High setup and validation time	Lighting/occlusion/noise; dataset shift & domain adaptation; code/model versioning	High	(183, 185, 205)

### 4.1 Traditional assessment methods

#### 4.1.1 Gait analysis

Under the combined influence of peripheral inflammation and central sensitization, spontaneous pain can manifest as anatomically specific gait alterations (118, 119). Such functional changes more closely resemble the disability component of chronic pain, because shifts in weight bearing, stance phase, and interlimb coordination can reflect ongoing discomfort rather than a brief withdrawal reflex. Gait analysis is also well suited for evaluating candidate therapies, as sedative or motor suppressive drugs typically do not restore normal walking and therefore are less likely to yield false positive analgesic signals; accordingly, preclinical gait endpoints have shown good reproducibility across studies (120). However, precisely because gait integrates both peripheral drive and supraspinal control, it is vulnerable to confounding by motor impairment, sedation, anxiety, and compensatory strategies, which may not align between rodent models and human patients. Recent NEP focused systematic reviews and syntheses of pain suppressed behaviors therefore recommend interpreting gait as “function under pain” and combining it with measures of comorbidity and affective motivational domains to strengthen translational inference (62).

Common approaches quantify paw-print morphology (e.g., print area/intensity), spatiotemporal parameters (stride length, stance phase), and related metrics to infer pain intensity and quality (121, 122), and are widely used in spontaneous-pain research. The main limitation is limited specificity: gait alterations can be confounded by non-pain factors such as changes in locomotor pattern or environmental stress (123). Another frequently used method scores hind-paw use/weight-bearing after walking—most common in bone-cancer pain and acute inflammatory models—typically with a four-point scale for the affected limb (124). This approach requires minimal equipment and is easy to implement but is highly observer-dependent and time-consuming, limiting its utility in large, high-precision studies. To address these gaps, Krug et al. developed advanced dynamic weight bearing (ADWB), which leverages software innovations to quantify pain severity and has shown greater sensitivity and reproducibility in intra-articular therapy and inflammatory-pain assessments (125). Dent et al. also used ADWB in mice to evaluate ultra-acute hypersensitivity induced by allyl isothiocyanate (AITC); objective, non-reflexive quantification of “loading/unloading” improved measurement objectivity (126). A related continuous hindlimb-use scoring paradigm is commonly applied in peripheral neuropathic models such as sciatic nerve ligation; it performs well for chronic pain but is less sensitive to acute nociception (127).

By comparison, the CatWalk system combines automated video capture with computer-assisted analysis to detect subtler gait perturbations and has been widely adopted across central and peripheral disease models (119, 128). It provides real-time, condition-specific quantification of multiple gait parameters (129, 130). Advantages over traditional methods include: (1) integrated multidimensional metrics—symmetry, stride length, cadence, velocity—which substantially increase resolution for pain-related phenotypes (122); and (2) objective quantification of spontaneous pain and hyperalgesia, improving interpretability and reproducibility (131, 132). Nonetheless, consistency can be suboptimal in certain contexts; for instance, sensitivity is limited in mild mono-arthritis (133). Using well-matched controls (e.g., body weight/length) can mitigate this issue. Cross-platform comparisons are also complicated because systems such as CatWalk, DigiGait, and TreadScan extract non-identical parameter sets.

Effectiveness also varies by rodent strain. Static weight-bearing is highly informative for hind-paw pain in rats but can be challenging in mice. Strain- and sex-specific differences are well documented (133, 134); for example, in C57BL/6 mice, females exhibit more spontaneous pain behaviors than males in chronic degenerative joint pain models (135). So controls should be matched by strain and, when feasible, strain specific reference ranges should be used when interpreting pain related gait changes. All gait-based techniques require standardized behavioral training prior to data collection. Acclimation to handlers and the test environment can markedly influence pain-related performance. Commercial gait platforms such as CatWalk typically require greater equipment investment than direct video recording (119), but they provide automated extraction of multidimensional gait parameters (136), thereby facilitating standardized measurement and higher throughput. In practice, individual acquisition runs are relatively brief, whereas the primary time burden is front loaded to pretest habituation and training and to obtaining a sufficient number of valid trials per animal. Consequently, gait based approaches generally represent a high equipment cost but comparatively low manual scoring burden (137).

#### 4.1.2 Facial expression

Similarly, grimace based pain scoring can capture animals' natural pain responses in the absence of external stimulation. By indexing a spontaneous, affect laden expression of pain, it aligns more closely with clinical pain constructs that rely on central integration, and thus provides a cross species compatible readout of spontaneous pain (138). These methods classify and score pain-linked facial action units. The grimace scales were introduced in rodents over a decade ago and have since been adapted to at least ten mammalian species (139–141).

The Rat Grimace Scale (RGS) (142) and Mouse Grimace Scale (MGS) (143) quantify facial pain by rating parameters such as eye fissure (orbital) narrowing, ear position, nose/cheek bulge, and whisker orientation. These tools exhibit high sensitivity and good test–retest reliability across postoperative, neuropathic, and visceral pain models and are notably effective for detecting the effects of low-potency opioids (144–146). Compared with evoked assays, grimace scales avoid additional injury, suit designs that must minimize intervention, require only a camera and frame-capture software, and demand no animal training—reducing non-pain confounds.

Limitations remain. In postoperative (147, 148) and inflammatory models (149), RGS/MGS are discriminative, yet facial changes often resolve within hours to ∼48 h, lagging the underlying pain course and constraining chronic tracking (150) Some individuals with chronic pain—preclinically and clinically—show minimal overt facial discomfort (151, 152). Specificity can also be context-dependent: RGS tracks dynamic pain changes in colitis (70) but is less responsive in chemotherapy-induced mucositis (146). As with other spontaneous pain measures, grimace based scoring shows both sex differences, often with stronger effects in males than females, and strain dependent variability. For example, one study reported a graded pattern of scores among male mice, with C57BL/6 lower than CD 1 and C3H/He, and a similar strain related gradient was also observed in females (153). In addition, grimace scoring typically relies on manual observation, making it time consuming and susceptible to observer related subjectivity. Reviews of rodent grimace scales indicate that this approach is useful for identifying ongoing pain states and for monitoring analgesic effects, while also emphasizing its sensitivity to contextual factors and methodological choices (154).

To address these issues, computer vision and AI are increasingly integrated into pain assessment. Automated scoring and machine-learning platforms promote inter-lab standardization while enabling parallel appraisal of affective states. Tuttle developed an automated MGS (aMGS) using a deep neural network (InceptionV3), achieving accuracy comparable to human raters (155). Similarly, Arnold et al. reported an automated RGS with ∼97% precision for action-unit detection and reliable prediction of composite RGS scores (156). Parallel advances in human pain assessment include deep-learning systems for postoperative/clinical videos that can partially distinguish genuine from posed pain expressions (157, 158); Kim and colleagues combined Transformer architectures with dynamic video encoding to improve temporal resolution in facial pain tracking (159). Despite continued progress, acquiring, curating, and analyzing high quality facial imagery remains labor intensive, which limits scalability for rapid turnaround and large scale screening. Overall, the method has a low equipment barrier and short per session acquisition time, but its primary bottleneck is the time and personnel required for manual scoring. Automated approaches generally shift effort upstream, requiring greater initial investment in data preparation and model development, while substantially reducing downstream scoring and analysis workload (160). However, grimace remains an indirect readout of endogenous inhibition and is best paired with assays that explicitly probe descending control or pain relief–driven preference, such as CPP or CPA.

Regarding reliability, systematic reviews indicate strong inter- and intra-rater agreement in rodent models, with weaker evidence in other species (e.g., rabbits, pigs) (150). These constraints have accelerated multimodal strategies—for example, combining noninvasive behavioral indices (nesting, burrowing) with grimace scales (161), or synchronizing facial metrics with neuroimaging, electrophysiology, and thermoregulatory readouts to establish convergent validity (162, 163). Emphasizing noninvasive techniques reduces stress and aligns with the 3Rs in the development of pain-assessment systems (156).

#### 4.1.3 Affective–motivational

In recent years, conditioned place preference/aversion (CPP/CPA) has been used increasingly to interrogate spontaneous pain in rodents. The core logic is to measure preference or avoidance shaped by tight pain–context associations: during ongoing pain, an intervention that relieves pain becomes associated with a specific context and yields CPP; conversely, contexts paired with pain are avoided (CPA). CPP/CPA directly probes the affective and motivational dimensions of pain, and this conceptual framework has been validated in systems neuroscience. Mechanistically, pain aversion and the rewarding value of pain relief are encoded by midbrain–limbic circuitry. Accordingly, CPP/CPA can quantify otherwise hard-to-observe ongoing spontaneous pain rather than mere changes in reflexive responses, thereby better approximating the true disease burden of chronic pain (164, 165). The apparatus is relatively standardized and does not require expensive imaging systems, but it does require compartmentalized chambers, careful control of contextual cues, and behavioral tracking. Because much of the procedure remains manual, the time burden is substantial, typically involving multiple days of habituation, conditioning sessions, and testing (166).

In practice, CPP is frequently deployed for mechanism validation under precise interventions. For example, selective blockade of afferent transmission from the injured segment by dorsal root ganglion field stimulation (GFS) reduces spontaneous dorsal horn firing in nerve-injury models and produces analgesia-induced CPP, thereby closing the causal chain of “spontaneous pain → dorsal horn hyperexcitability → reward-like behavior” (167). Inhibiting T-type Ca^2+^ channels (Cav3.2) in peripheral sensory neurons alleviates both spontaneous and evoked pain (168). CPP also gauges abuse/reward liability by testing whether a candidate analgesic elicits CPP in the absence of pain relief, separating true analgesia from drug seeking (169). Recent studies further report sex differences in CPP readouts in some trigeminal neuralgia models, underscoring the need to incorporate sex in design and interpretation (170). Whereas CPP emphasizes negative reinforcement within VTA/NAc circuits, CPA engages cortical–limbic pathways (e.g., ACC) and does not rely on relief-reward as an indirect proxy; it is thus well suited to visceral-pain affect. In bone cancer pain, manipulating TrkB, NR2B, or ERK–CREB selectively weakens avoidance without altering mechanical thresholds, indicating that CPA maps more closely onto pain's affective dimension (20). Meta-analytic summaries of CPA emerging 1–2 weeks after inflammatory insult show time sensitivity and reproducibility for the affective facet of spontaneous pain (171).

CPP and CPA capture the affective and motivational dimension of pain and can be linked to circuit level readouts, which supports their cross species potential and translational relevance. In the context of endogenous pain modulation, descending inhibition or facilitation can shift the net aversiveness of ongoing pain and the value of analgesic relief, thereby changing preference or avoidance strength. This makes CPP and CPA, to some extent, a functional readout of central regulation rather than a mere surrogate of peripheral sensitivity, and such central modulatory differences are often more pronounced in chronic pain states. Consistent with this view, conditioned pain modulation in humans is commonly considered the behavioral counterpart of diffuse noxious inhibitory controls in rodents and is used to probe descending inhibitory function (172). Cross species credibility is further strengthened by circuit evidence. In mice, expectation and higher order appraisal can recruit specific cortical pathways to produce analgesia, a core feature of central modulation also emphasized in human studies (173). Human molecular imaging and fMRI work on the nucleus accumbens and related reward circuitry likewise supports translational mapping between pain aversion and relief related reward (174, 175). In clinical practice, CPM is typically operationalized using dynamic QST paradigms, in which changes in the perceived intensity of a test stimulus during a spatially remote conditioning stimulus are used to index descending inhibitory function. By contrast, DNIC in animals is often assessed under anesthesia or electrophysiological settings, with suppression of dorsal horn neuronal activity or spinal reflex outputs as the primary readout (176). Notably, although both paradigms capture “pain inhibits pain” related descending modulation, CPM is more susceptible to higher order influences such as attention, expectancy, and affect, and therefore is not fully equivalent to DNIC (172). Moreover, contextual preference forms only when animals are in an aversive, spontaneous-pain state and analgesia is paired with a specific context, conferring greater specificity relative to short, acute evoked pain. Finally, CPP and CPA can be coupled with electrophysiology, *in vivo* imaging, and circuit manipulations to strengthen mechanistic inference. For example, lactate signaling in anterior cingulate cortex astrocytes has been detected during the formation and retrieval of visceral pain CPA (177) and fiber photometry and single unit recordings have reported VTA, nucleus accumbens, and anterior cingulate cortex activity aligned to CPP or CPA expression (178). Together, these features make CPP and CPA well suited for testing interventions that target central modulation by asking whether they truly reduce the aversiveness of ongoing pain and the value assigned to analgesic relief, supported by convergent multimodal evidence.

As with other assays, CPP/CPA is influenced by sex, strain, and stress (179). Classic methodological reviews also note confounds from individual differences in learning/motor ability and from apparatus variability (180). In line with recent trends, these limitations can be mitigated by: (1) high-throughput video co-acquisition of spontaneous behavior to integrate CPP/CPA with markerless pose estimation and interpretable pain-phenotype extraction (181); (2) synchronizing CPP/CPA with *in vivo* calcium imaging/electrophysiology to record circuit activity in real time (e.g., fiber photometry of ACC projections and NAc dopamine) (165); and (3) ML-based feature extraction with individualized thresholds to unify high-throughput video, calcium, and electrophysiology, thereby reducing context/motor confounds and improving inter-lab reproducibility and translational value.

### 4.2 AI enabled multimodal assessment

In current preclinical work, traditional assays predominantly rely on stimulus-evoked reflex thresholds. These readouts are specific but neglect domains most relevant to the clinic—spontaneous and affective–motivational dimensions—and therefore have intrinsic limitations for detecting chronic, ongoing pain. Low-intervention approaches (e.g., home-cage monitoring of spontaneous behavior, facial grimace scoring) offer broader coverage but with constrained specificity and more complex interpretation. Reproducibility is further hampered by contextual factors and inter-individual variability.

#### 4.2.1 AI automation of behavioral readouts

For evoked assays such as von Frey, Hargreaves, and hot-plate tests, the key bottlenecks are bias during stimulus delivery/observation and low throughput. Dedek designed an integrated, computer-controlled platform for thermal and mechanical stimulation and trained a neural network for automated measurement—while also initiating video-based capture of spontaneous behavior (75). Pairing high-frame-rate video with machine learning enables millisecond-scale, unbiased kinematic analysis of paw withdrawal (182). Three-dimensional, multi-camera capture with keypoint tracking mitigates the “reaction-only, context-blind” limitation of evoked tests. Although generic clustering may mistake high-frequency jitter for target actions, a keypoint-MoSeq pipeline can discover behavior modules from keypoints without supervision (e.g., rapid sniffing) (183). In diabetic neuropathy (DN) models, 3D behavior analysis combined with unsupervised learning (MoSeq) yields finer phenotyping and treatment sensitivity (184); MoSeq likewise strengthens gait analysis by reducing dependence on running speed and training, improving cross-lab reproducibility. Automated micro-movement detection further reduces observer bias: the Facemap framework couples keypoint tracking with a deep encoder to extract eyelid aperture, whisking, and nose–mouth micromotions from video, delivering high-throughput grimace analysis while predicting neural activity (185). Relatedly, DeepLabCut-based pipelines annotate eyelid distance/palpebral fissure to quantify mouse facial pain (186). Butler and colleagues fluorescently labeled distal limbs and used multi-view, variable-illumination video to auto-generate large training sets, improving generalization to fine movements—a strategy that may remedy weak whisker-unit detection in grimace scales (187). These modern approaches entail substantial upfront costs, including specialized hardware, computational resources, data annotation, and model training. Once the pipeline is established and validated, however, the per animal manual workload drops markedly, enabling greater scalability (188).

#### 4.2.2 Translational robustness: confounders, validation, reporting

It is important to note that automation and higher apparent accuracy primarily improve measurement consistency and do not necessarily yield more clinically translatable conclusions or deeper mechanistic insight; their value still depends on external validation and systematic control of confounding factors. To mitigate these risks, the development of modern assessment pipelines should prioritize interpretable pain relevant readouts, such as the burden of ongoing spontaneous pain and its affective and motivational dimensions, while concurrently recording potential confounders including locomotor capacity, sedation, and stress to enable separation during analysis. Models should then be externally validated and calibrated across strains, laboratories, and acquisition platforms, with their intended scope of use clearly defined. Reporting can follow frameworks such as TRIPOD plus AI (189), and risk of bias and applicability can be assessed using tools such as PROBAST plus AI (190). Finally, converging behavioral AI readouts with central network endpoints, for example fMRI, or circuit level measures can help distinguish phenotype level association from mechanistic concordance, thereby turning more objective measurement into more reliable translational inference.

#### 4.2.3 fMRI as a central translational endpoint

Functional MRI offers a clear advantage for discussing pain processing and its translational relevance. It can generate comparable readouts of distributed brain networks in both humans and rodents, allowing endpoints at the level of central processing rather than limiting inference to behavioral reflexes. In a landmark PNAS study, Hess et al. (2011) showed that neutralizing TNF α markedly reduced nociception related activity in the thalamus, somatosensory cortex, and limbic regions within 24 h, indicating that neuroimaging can directly link a molecular intervention to brain level pain processing and providing evidence for central mechanisms underlying rapid analgesic effects (191). More recent rodent fMRI work further demonstrates that distinct pain conditions can produce dissociable patterns of central processing with translational implications. For example, Kreitz, Pradier, Segelcke, combined fMRI with network analysis and classification in incisional and inflammatory pain models to identify condition specific hypersensitivity related network signatures (192, 193). Their results suggest that pain states involve not only changes in sensory pathways but also coordinated alterations in aversion related processing and components linked to descending modulation. The authors further proposed that such signatures could support disease relevant brain biomarker discovery and drug target engagement evaluation, thereby improving the testability of translation.

Building on this framework, incorporating additional modalities can further enhance automated assessment and provide a more complete evidence chain for translational validation of central mechanisms. Ultrasonic vocalizations (USV) index affective state (low-frequency, aversive; high-frequency, positive); combining USV with deep learning may reduce confounds from individual differences in motor capacity (194). Infrared thermography (IRT) non-contactly tracks skin temperature and microcirculation and is widely used to monitor inflammation (195); ML-based denoising, super-resolution, and multi-feature fusion can incorporate such physiological changes into holistic pain scores (196). On the device side, piezoelectric sensing was used as early as 2007 to analyze respiration during sleep with automated pattern recognition (197). Recent open-platform systems built on ultra-sensitive piezos detect the slightest movements—even heartbeats—and integrate video with time–frequency decomposition, clustering, and machine-learning analytics to provide noninvasive behavioral assessment (198). Inevitably, increasing technical complexity introduces new challenges in standardization, data integration, ground-truth labeling, and scalability that must be addressed for robust translation. Combining multimodal measures with central network readouts such as fMRI, and benchmarking them under standardized protocols with consistent metadata capture and external validation, will help multimodal AI pipelines mature into reusable and generalizable pain assessment systems.

### 4.3 Strain effects and generalizability

Strain is a major source of biological variability in pain research and can strongly shape the apparent performance of behavioral assays. Across evoked stimulation paradigms, spontaneous pain model induction, and behavioral assays for pain, animal strain remains a pervasive and nontrivial confounder. In mice, large scale comparative work as early as 1999 demonstrated substantial differences among inbred strains across multiple commonly used nociceptive and antinociceptive endpoints (199). More broadly, rodent strains exhibit systematic variation in baseline nociceptive responsiveness, affective and locomotor traits, learning and memory, and drug sensitivity, which can markedly influence the reliability and reproducibility of pain assessment even under otherwise identical experimental procedures (2). Accordingly, within strain comparisons should be prioritized in pain research. When the goal is broader generalizability, key findings can be replicated in at least one additional strain. For cross strain studies, we recommend prespecifying strain as a biological variable, harmonizing protocols and metadata capture across laboratories, and modeling strain effects through stratified reporting and mixed effects analyses. These considerations also provide a strong rationale for developing cross strain databases (200).

## 5 Conclusion

Successful clinical translation in pain research hinges on aligning the research question, the animal model, and the clinical mechanism–phenotype match. Contemporary clinical paradigms have moved beyond simple “stimulus–reflex” thinking toward multidimensional, holistic assessment frameworks (206).

By contrast, preclinical studies still largely quantify suppression of behavior after noxious stimulation in otherwise healthy animals—effectively testing whether protective responses are blocked. This misalignment helps explain why many “effective” preclinical analgesics fail in patients. Crucially, what burdens patients most is spontaneous, persistent pain, not brief evoked hypersensitivity (66, 207). Thus, improving translational efficiency starts with modeling and endpoints that are isomorphic with clinical constructs.

To this end, three challenge domains must be addressed head-on: (1) Model–population mismatch and limited external comparability—induction strategies, surgical techniques, and biological variables (strain, sex, age) introduce substantial batch variability; (2) Endpoint monism—overemphasis on reflex thresholds while neglecting spontaneous behaviors and affective/autonomic dimensions; (3) Bias and reproducibility—observer dependence, and environmental/handler stress that markedly shift outcomes.

Priorities should include adopting modeling strategies congruent with clinical contexts; pre-registration (in multicenter designs) of covariates such as strain/sex/age, circadian phase, housing and equipment, and operator; and external validation using resources like the Mouse Phenome Database (208) and standardized von Frey/Hargreaves protocols (209). Within multicenter frameworks, sex differences (210), housing/enclosure features (211), and experimenter effects (130) should be treated as explicit variables.

**Figure 3:**
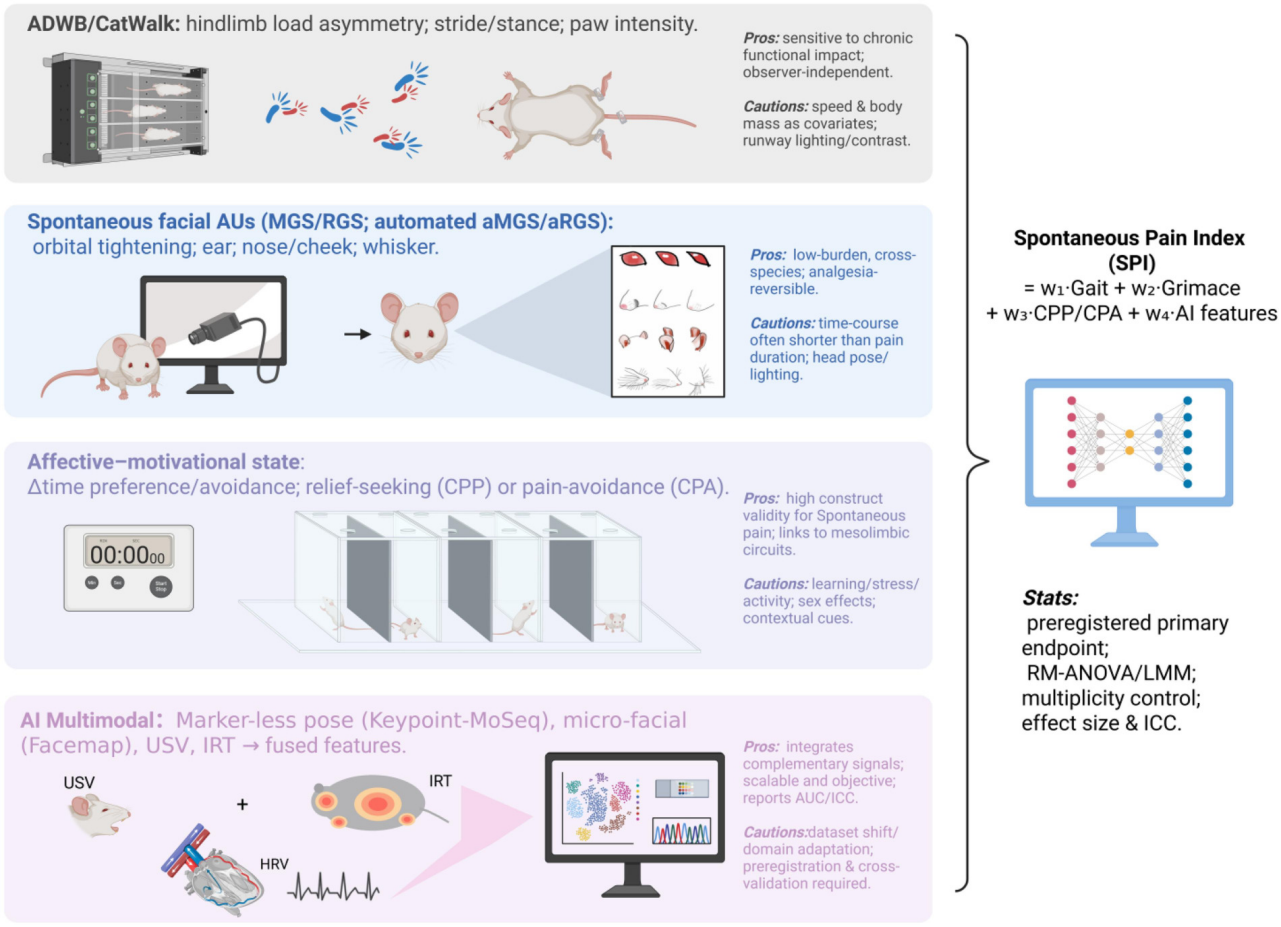
From traditional to modern spontaneous-pain assessment. Gait (functional), facial grimace (somatic-affective), and CPP/CPA (affective-motivational) provide complementary non-evoked readouts. An AI multimodal layer fuses these measures into a preregistered Spontaneous Pain Index (SPI). Arrow notes indicate each panel's strengths and cautions; dashed gray arrows denote confounders/controls; bold red arrows indicate contribution to SPI.

On the assessment side, spontaneous-pain indices should be foregrounded: spontaneous behaviors (nesting, burrowing, load transfer, sleep continuity), facial expression (RGS/MGS), affective–motivational readouts (CPP/CPA), and autonomic/physiological signals—HRV (heart-rate variability), USV (ultrasonic vocalizations), and IRT (infrared thermography). Use linear mixed models and report effect sizes and ICC (intraclass correlation coefficients) to strengthen objectivity and reproducibility. To strengthen alignment with human studies, we propose a two-layer translational bridge. The first layer links human QST and CPM to animal readouts: human QST measures thermal and mechanical detection and pain thresholds, which map to Hargreaves and von Frey; human temporal summation aligns with dorsal-horn central-sensitization behaviors and electrophysiological endpoints; and human CPM can be related to experimental manipulations of descending inhibition as well as the relief valuation captured by CPP and CPA. The second layer adds a complementary central-network endpoint. Because fMRI can quantify distributed brain-network activity in both humans and rodents, it offers a practical way to test whether candidate interventions engage conserved central circuits and to assess target engagement beyond reflex behavior alone (212). Finally, integrate video pose, facial micromovements, USV, IRT, and HRV into a shared, open AI pipeline—publishing algorithms and code—to establish, as illustrated in Figure 3, a reusable, externally verifiable Spontaneous Pain Index (SPI) benchmark.

Recent interdisciplinary advances underscore feasibility. Bohic et al. used supervised and unsupervised machine-learning tools for 3D pose analysis, identifying movement modules that discriminate pain states (213). Similarly, deep-learning–based feature-fusion models achieve high accuracy in facial-expression assessment, laying a foundation for multimodal data integration. In the clinical arena, intelligent pain-management systems in cancer care—combining real-time data capture with AI-driven analytics—have improved precision control of pain and reduced the incidence of breakthrough pain (214). Collectively, these developments highlight the potential of cross-scale, cross-domain data integration and the promise of intelligent pain-assessment systems. Advanced under rigorous metadata standards, benchmarking, and external validation, these directions are poised to enhance the translational value of preclinical research and accelerate the practice of precision pain medicine.
